# 

**Published:** 1806-02

**Authors:** 


### TO CORRESPONDENTS.

Communications are received from Dr. Cuming, Mr. Simpson, Mr. Hall, Mr. Clarke, Mr. Namlat, and Antipeculator,

